# Nonverbal Oro-Motor Exercises: Do They Really Work for Phonoarticulatory Difficulties?

**DOI:** 10.3390/ijerph19095459

**Published:** 2022-04-29

**Authors:** Pablo Parra-López, Marina Olmos-Soria, Ana V. Valero-García

**Affiliations:** Department of Developmental and Educational Psychology, Faculty of Psychology, University of Murcia, 30100 Murcia, Spain; marolmos@um.es (M.O.-S.); vanesavg@um.es (A.V.V.-G.)

**Keywords:** articulation disorder, nonverbal oro-motor exercises, intervention in difficulties in articulation, practice-based evidence, phonetic disorder, childhood

## Abstract

Articulation disorders are deficiencies in the realization of speech sounds unrelated to organic or neurological disorders. Over the last decade, there has been a debate on the efficiency of non-verbal oro-motor exercises, which are orofacial movements programmed and organized in an intentional and coordinated way to control lips, tongue, and soft palate muscles. Of the 122 children evaluated, 52 presented articulatory difficulties. An intervention with nonverbal oro-motor exercises was applied, and children were again assessed following treatment. The results showed no differences between the experimental and control groups, either in the number of sounds that improved after this period or in the severity of difficulties (we categorized those with articulation difficulties in two to six sounds as ‘medium’ and those with difficulties in articulating more than seven sounds as ‘severe’). These results indicated that nonverbal oro-motor exercises alone are not efficient for intervention in difficulties in the realization of sounds in 4-year-old children.

## 1. Introduction

It is common for children with typical development to err in the realization of sounds in their speech development process, but these difficulties should give way to understandable speech around the age of 3 years, according to the DSM-5 [1]. Articulatory phonetics considers how articulatory organs produce language sounds varying in four characteristics: the place of articulation, manner of articulation, nasality–orality, and voiced–voiceless [2,3].

Articulation disorders are difficulties in phoneme acquisition, i.e., difficulties in correctly pronouncing some phonemes or groups of phonemes, which can often cause unintelligible speech in the child [4,5]. In Spain, these deficits have traditionally been labeled as ‘dyslalias’. Thus, authors such as [6] defined ‘dyslalia’ as a disorder in the articulation of one or more sounds that are produced later than expected in typical development. In this regard, functional articulation disorders should be understood as a deficit in the pronunciation of phonemes produced by a delay in motor maturity, i.e., by an inadequate function in peripheral organs of speech, without organic or neurological lesions [4,7]. Other authors included articulation disorders within ‘phonological disorders’, following the psycholinguistic paradigm [2,8] and the new APA conceptualization [1]. Following this approach, phoneme acquisition is based on phonological knowledge and the ability to coordinate the movements of articulatory organs such as lips, tongue, and soft palate, as well as breathing and vocalization of speech. Hence, alterations in phonological production are due to problems in the phonological knowledge of speech sound or to problems in the ability to coordinate speech movements, so the phonological disorder includes both phonological and articulation deficits [1].

From this perspective, phonetic and phonological disorders are considered to be different both in etiology and intervention [9,10]: the former are speech deficiencies where the phonetic aspect is affected because of a delay in the development of articulatory aspects of a functional nature, while phonological disorders are a speech disorder wherein difficulties are observed in auditory discrimination and phonological programming—placing phonemes in the right order within the word [11,12]. When continuing the differentiation of these two concepts, phonetic disorders are characterized by error stability (i.e., they do not improve with repetition), children are usually aware of their mistakes, and these may not appear in their writing. On the other hand, phonological disorders are characterized by error instability; they improve with repetition, children are usually unaware of their mistakes, and these are commonly reflected in their writing [9]. Children with phonetic disorders make articulation errors both in the repetition of syllables and sounds as well as in isolated words and in the sentence context, as they lack the necessary motor coordination and accuracy in phonoarticulatory organs, and therefore, errors are systematic and independent of the phonetic context. However, in children with phonological disorders, the production of the isolated sound is usually correct, and the error pattern is not systematic owing to the fact that these errors vary in relation to the phonetic context, e.g., a child can have a correct production of the phoneme /k/ in the word ‘car’ but substitute /t/ for it in the word ‘chocolate’, producing ‘chotolate’ [12,13]. Furthermore, in most cases, the child presents both types of errors [12,13,14]. The most problematic phonemes are those requiring a more precise motor accuracy, such as phonemes /r/, either simple or multiple, and the consonant groups [15,16]. Since, in order to be pronounced, they require fine coordination and control of the muscular groups involved [17], these can be long-lasting, as found in Preston’s study [18] with a group of children with articulation disorders whose average age was 4.6 years and whose deficiencies lasted for 4 years.

However, other authors have claimed that there should be a division in the denomination of phonological disorders to differentiate articulation disorders. These include those substitutions or distortions of isolated sounds in all phonetic contexts during imitation, elicitation, or spontaneous speech tasks [19]. Articulation disorders are classified as developmental, auditory, organic, and functional [4,12,20]. Among the different causes, studies have related them to memory problems, as well as to immediate auditory memory, immediate visual memory, and motor speed, together with attention deficit [21]. Some speech therapy textbooks mention the following as etiological factors: the lack of control of fine motor skills (articulation of speech requires large motor skills), deficits in auditory perception and discrimination, low linguistic stimulation, bilingualism, psychological factors (overprotection, traumatic situations, etc.), atypical swallowing, intellectual deficiency, etc. [22,23,24]. In most cases, these factors are not present in isolation, but several appear together [12,25]. Articulation problems can affect the socio-emotional development of the child so that they turn shy, anxious, afraid to speak, and aggressive, and there is a decrease in social relations resulting in social isolation [26,27]. Recently, as an integration of both (phonetic and phonological) perspectives, [28] proposed the concept of ‘articulatory gesture’ based on the Articulatory Phonology Model [29], which, in turn, lies in Dynamic System Theory. They propose that the development of speech sounds in children is achieved through the development of speech perception but also the maturation of articulatory gestures. They consider that ‘all levels involved in speech production are part of a complex system with processing stages that are highly integrated and coupled at different time scales’. 

As for intervention, some authors have considered common speech therapy practices for these difficulties, directed at auditory discrimination, orofacial motricity, and breathing and blowing. Once the sound is achieved, it should be integrated into speech by repetition and generalized in the child’s elicited language (for example, with images). Finally, it should be integrated into their spontaneous language [14,15]. The causes of articulation disorders are multifactorial; therefore, their treatment must also be multidimensional. For this reason, we suggest analyzing the efficiency of intervention techniques in an isolated way, without meaning that the treatment should only be based on nonverbal oro-motor exercises. In the psycholinguistic model, these difficulties are considered to be ‘phonological simplification processes’ or phonological processes that are conceptualized in a set of mental operations that children use to simplify adult speech [30,31], i.e., to simplify the adult sound by adapting it to their possibilities of expression. Some authors have offered interesting discussions on this topic [32,33]. These processes can be grouped into three categories: (1) processes related to the syllabic structure, where the child simplifies any syllable to adapt it to their way of speaking according to their developmental level; (2) substitute processes, when the child changes a contractive segment for a different one that is easier to pronounce; and (3) assimilatory processes that appear when children make a sound similar to another in the same word—for example, ‘totolate’ for ‘chocolate’ [2,8,30,31,34].

Other authors have included nonverbal oro-motor exercises in the treatment of articulation disorders from the phonological standpoint [31]. When difficulties are articulatory and perceptive, these authors proposed intervention with two aims; the first focused on perceptual development with exercises associating sounds and words with related images, and the second pursued phonoarticulatory elicitation. This includes the initial assessment of the session, nonverbal oro-motor exercises, modeling, training in location placement, and shaping and reading of words and phrases. The debate is still ongoing regarding the use of nonverbal oro-motor exercises, which are understood as important training to perform and articulate learned movements with the tongue, lips, jaw, and facial gestures with the aim of producing phonemes and words [35]. According to the Evidence-Based Practice (EBP) model of the ASHA, we should integrate empirical evidence from research with the professional experience of speech therapists and the characteristics of the patient in the process of making clinical decisions. Thus, some authors have claimed that nonverbal oro-motor exercises are still an ideal treatment for articulation disorders [36,37,38,39]. Indeed, there are few studies in speech therapy scientifically supporting interventions with nonverbal oro-motor exercises [13]. In contrast, several studies have empirically analyzed relations between fine motricity and language developmental disorders and considered nonverbal oro-motor exercises not useful for speech disorders, and they were dubious regarding their efficiency [40,41,42,43,44]. Thus, Lof [41,42] reviewed interventions with non-speech oral movements and concluded that no evidence exists that these exercises improve the child’s speech, although he also pointed out that because a group of techniques is used at the same time as the nonverbal oro-motor exercises, it is, therefore, difficult to know the precise role of these movements in the child’s improvement of sound realization. In addition, this author questioned the scientific rigor of these studies and stressed the need for research using individual treatments to prove their effectiveness. In line with these authors, a study was carried out in the US where speech therapists were asked whether they used nonverbal oro-motor exercises and why with results showing that 85% of American professionals considered them useful for the intervention in the production of speech sounds [43]. Similarly, Furlong [45] found that the traditional articulation approach in conjunction with minimal pairs was the most common therapy used for speech sound disorders by the Australian SLPs they interviewed. Nevertheless, when American University speech therapists were asked that same question, the results showed that 25% of professors recommended nonverbal oro-motor exercises, and 75% did not, warning that their effectiveness is in question [46,47].

In Spain, some authors consider nonverbal oro-motor exercises an effective tool [35,48], and some even use them when taking the phonological approach [31]. However, other authors have claimed that nonverbal oro-motor exercises are not useful [8,11]. In the most recent review on the efficiency of nonverbal oro-motor exercise programs and verbal treatments based on phonemes, syllables, and words, Ygual-Fernández [47] concluded there are no arguments to support the use of nonverbal oro-motor exercises.

Therefore, in this study, we analyzed the efficiency of nonverbal oro-motor exercises for the acquisition of phonemes in children with articulation difficulties at 4 years of age. A second aim was to determine which phonemes or groups of phonemes nonverbal oro-motor exercises are more efficient. The third objective was to analyze whether the benefits are the same regardless of the severity of the difficulties.

Thus, our first hypothesis is that there will be differences between the experimental and control groups in the number of difficulties in the realization of sounds once the intervention with nonverbal oro-motor exercises is implemented. The second hypothesis is that nonverbal oro-motor exercises will improve in a differential way the production of the different sounds. The third hypothesis is that nonverbal oro-motor exercises will be more efficient in addressing more severe difficulties in realizing sounds.

## 2. Materials and Methods

### 2.1. Participants

Authorization was requested from the center and from the parents of students without disabilities in the 2nd year of Early Childhood Education in two preschools in Murcia, one in the city center and another on the city outskirts. Both schools are part of an urban area. Of all the parents, 99% approved of their children taking part in the study, as they were offered a report on their children’s speech development at the end. The sample comprised 122 participants, 60 boys (49%) and 62 girls (51%) with a mean age of 4 years and 7 months (*M* = 55, 28 months, range = 11, minimum = 50 and maximum = 61). The children evaluated had a typical development and belonged to families with a medium socioeconomic level. For this study, 4-year-olds were chosen, as they are usually the ones who simplify speech and have completed the phonological system of contrasts and the full development of their perceptual capacity [49] and also developed adequate motor skills to articulate the entire specific phonetic range of their native language [12].

Table 1 shows the distribution of participants in both centers. Children who had difficulties in producing two or more phonemes were divided into two types, depending on the number of difficulties in the realization of sounds. Thus, we classified difficulties as moderate when the children had problems with between two and six sounds and severe when they had difficulties with more than seven sounds. Half of the participants in each category were randomly distributed to the control or experimental groups. The number of children who had articulatory difficulties with more than two sounds was 55. One participant abandoned school during the study, and two children were eliminated for having a large number of articulation difficulties (more than 17 sounds) and in the assessment showed organic problems, although undiagnosed. Thus, the final number of participants was 52: 26 of these were assigned to the experimental group and 26 to the control group. As mentioned above, half of the children with moderate difficulties were assigned to the experimental group and the other half to the control group (randomly distributed by the other variables of sex and classroom). Similarly, children with severe difficulties were distributed between the experimental and control groups. There were seven experimental subgroups: five with four children and two with three children.

### 2.2. Measures and Procedure

Difficulties in the realization of sounds were assessed using the Induced Phonological Register [50]. This is a test based on the adult’s ideal model of pronunciation of the phoneme, and the sound not matching adult speech is considered an articulatory difficulty. The test evaluates induced and repeated language of children. They must name different drawings. In case of error, the child must repeat the word that the evaluator indicates. The material comprises 57 drawings of objects that cover the broad phonological spectrum of the Spanish language, although two more images (cross and dragon—‘cruz’ and ‘dragon’ in Spanish) were introduced to complete the consonant groups with /*r*/. The test was administered through a PowerPoint presentation to make the evaluation more dynamic. It took around 20 min to complete.

The test was administered individually in the speech therapy classroom. In order to familiarize the child with the situation and prior to the evaluation, the child was asked general questions. Upon completion, they were given drawings to color as a reward. All tests were recorded. The Induced Phonological Record was used in both the pretest and post-test. On completion of the test, children were asked to repeat isolated syllables, including phonemes that were incorrectly pronounced even in word repetition. A basic exploration with nonverbal oro-motor exercises was made (tongue moving up and down, around the lips, inside the mouth, toward the right and left sides of the mouth, etc.), noting whether pronunciation was correct, to eliminate the possibility of an organic deficiency. For this study, we were only interested in sounds that the child could not produce in either naming or repetition [51], as this indicated a disorder of a phonetic and not phonological nature. We decided to consider not only single phonemes but also the diphthongs, inverse phonetic groups, and consonant groups included in the test.

Once participants were evaluated and placed in different groups, the intervention with experimental groups began. This consisted of a series of 30 min sessions with nonverbal oro-motor exercises. It should be noted that these exercises produce fatigue, so intervention sessions should not be too long. These sessions occurred twice a week for 3 months in the school center, and each participant completed 24 sessions. Interventions were made in groups of three or four participants, depending on the subgroup, and in the soundproofed and specially equipped speech therapy room.

The intervention focused exclusively on the use of nonverbal oro-motor exercises, i.e., programmed orofacial movements organized in an intentional and coordinated form [52,53]. In order to choose the movements, we used the Ciceron Program for the acquisition and development of articulatory ability [54]. This program includes nonverbal oro-motor exercises for each phoneme, and we selected the most common movements in different phonemes. All children performed the complete set of exercises, regardless of the articulation difficulties presented (see Table 2). In all sessions, the exercises performed were noted.

The modeling of nonverbal oro-motor exercises with a mirror was used. Moreover, social and material reinforcement (drawings, stickers, etc.) were used to motivate children when the sessions were finished. After approximately 3 months, all participants in both groups were again assessed with the Induced Phonological Register.

### 2.3. Data Analyses

First, the frequencies of articulation difficulties in the pretest assessment were analyzed. In order to evaluate intervention efficiency, a repeated measures Generalized Linear Model (GLM) was chosen, taking the group as fixed factors and taking as response variables the number of sounds with articulation difficulties in the pretest and post-test assessments of experimental and control group participants. We used a repeated-measures model as there was a pretest and a post-test assessment. The data were analyzed using IBM SPSS Statistics for Windows (IBM Version 22.0, Armonk, New York, NY, USA).

## 3. Results

As mentioned earlier, this work aimed to study the usefulness of training in nonverbal oro-motor exercises in the intervention of articulatory difficulties.

In order to achieve this aim, it was necessary to first prove whether training with nonverbal oro-motor exercises improved the articulation of sounds and if so, to verify in which phonemes they are most effective.

**Figure 1:**
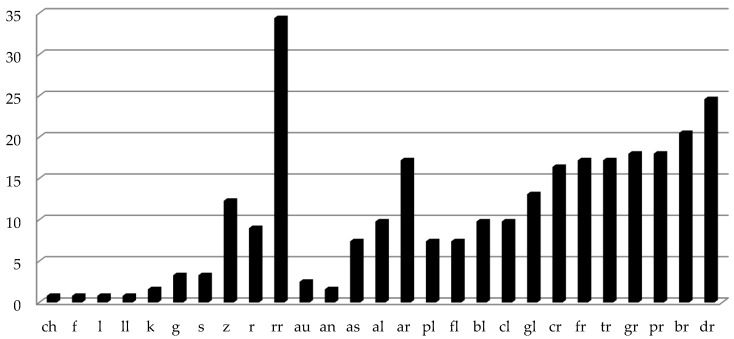
Frequency percentages of non-produced sounds in the pretest assessment.

The results showed that the nasal and occlusive phonemes are the first that all children acquire in the Spanish language since 100% emitted them correctly. Of the participants, 80% had fricative (/s/, /z/, /f/, /j/ and /y/), affricate (/ch/), and lateral (/l/, /ll/) phonemes. However, this percentage decreased to 66% for non-lateral (/r/ and /rr/) liquids, especially the multiple vibrant phoneme /rr/, as this was one of the most difficult to produce (see Figure 1). Most children had acquired diphthongs and inverse phonemes, while consonant groups are late to appear, and only 80% of assessed participants had acquired them.

Once children were evaluated, non-produced sound frequencies were calculated. In Figure 1, it is clearly observed that non-lateral liquid phonemes were the most difficult to pronounce (approximately 20%), as well as inverse alveolars (/al/, /ar/, /as/). Similarly, consonant groups con /l/ and /r/ were the most difficult for 4-year-olds to acquire. In order to analyze the effect of the nonverbal oro-motor exercises, a repeated measure analysis was carried out where the fixed factor was group (experimental versus control), and dependent variables were pre- and post-test evaluation. There were no significant differences following intervention between the experimental and control groups in the number of non-produced sounds *F*(1, 48) = 0.335; *p* = 0.565; *n_p_*^2^ = 0.007; *f*^2^ =.088; therefore, both groups improved, but not because of the oro-motor exercises (see Table 3). A post hoc power calculation was performed with G*Power. Given that an effect size of d = 0.4 is a good first estimate of the smallest effect size of interest in psychological research, we needed over 50 participants for a simple comparison of two within-participants conditions to run a study with 80% power (with *alpha* = 0.05). 

**Figure 2:**
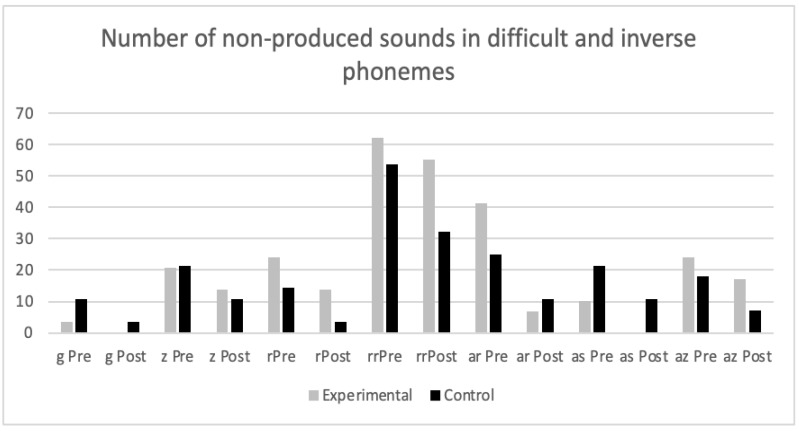
Number of non-produced sounds in difficult and inverse phonemes in the pretest and post-test in experimental and control groups.

**Figure 3:**
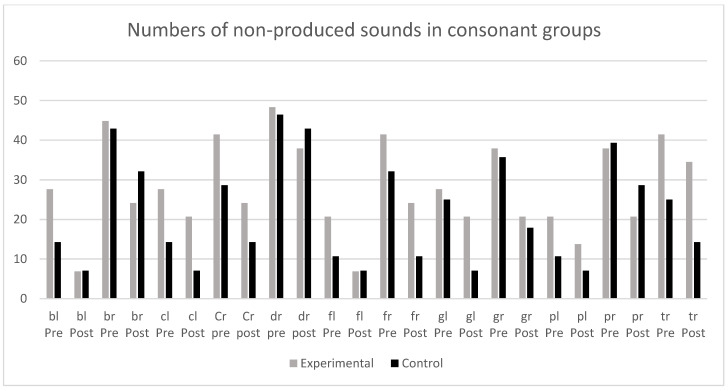
Number of non-produced sounds in consonant groups in the pretest and post-test in experimental and control groups.

In order to respond to another of our research aims, i.e., to determine in which sounds nonverbal oro-motor exercises were more effective, non-produced sounds were analyzed both in the initial evaluation and in the evaluation following intervention, i.e., the most difficult phonemes were considered (/s/, /z/, /r/, /rr/) as well as inverse phonemes (/an/, /ar/, /as/, /az/), and consonant groups with /l/ (/bl/, /cl/, /fl/, /gl/ and /pl/) and with /r/ (/br/, /cr/, /dr/, /fr/, /gr/, /pr/ and /tr/) (see Figure 2 and Figure 3).

In no case were there significant differences between the control and experimental groups, indicating that nonverbal oro-motor exercises were not especially efficient for difficulties with any specific kind of phonemes. Nor were differences found when phonemes were grouped in alveolar (/s/, /r/ and /rr/), inverse (/an/, /ar/, /as/ and /az/), fricative (s y z), and consonant groups with /l/ and /r/. We finally grouped non-lateral liquids (/r/ and /rr/) and again found the same result for non-significant differences between the experimental and control groups. In summary, the results showed that both groups (experimental and control) improved in the realization of sounds, regardless of intervention.

**Figure 4:**
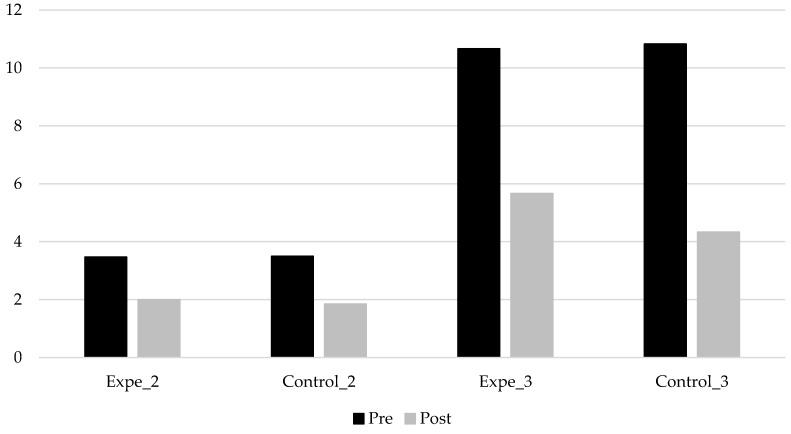
Mean of non-produced phonemes in the pretest and post-test depending on the severity of articulation difficulties (moderate difficulties: 2; severe difficulties: 3).

Our third aim was to analyze the efficiency of nonverbal oro-motor exercises depending on the severity of the articulation difficulties. Although there were significant differences in the realization of sounds in the pretest and post-test between participants with severe deficits and those with moderate deficits in a repeated measures GLM test (*F*(1, 48) = 86.528; *p* < 0.001; *n_p_*^2^ = 0.643; *f*^2^ = 1000), this effect was not due to the intervention, as an interaction between severity and treatment groups showed no significant differences (see Figure 4).

**Figure 5:**
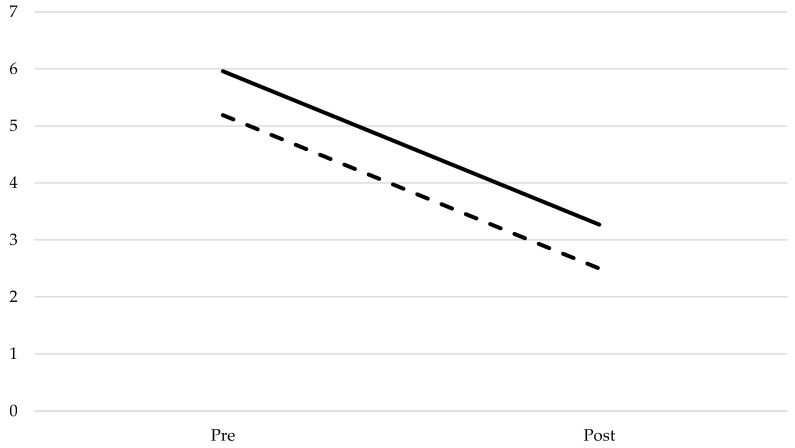
Mean of pre- and post-test non-produced sounds for the experimental and control groups.

In order to improve the results analysis, the means before and after intervention were compared following the repeated measures model. As reflected in Figure 5, there was a parallel linear decrease in the number of non-produced sounds for both groups. We observed that intervention did not have any effect on the participants‘ realization of sounds, as the differences between the pretest and post-test following intervention were not significant regarding the severity of participant difficulty measured by the number of non-produced sounds.

## 4. Discussion

The aim of this study was to analyze the efficiency of nonverbal oro-motor exercises for the articulation of sounds in speech development. Because they are generally used in professional practice, as mentioned in the Introduction and in the evidence-based practice model, it was useful to know if they are the most appropriate kind of intervention to undertake. The results showed no significant differences between experimental and control groups when nonverbal oro-motor exercises were used in children with typical development and non-produced sounds. After three months of intervention, both groups improved in their production of sounds, considering the number of phonemes in their speech in the pretest and post-test phases. Thus, our results indicated that these exercises are not useful for the acquisition of sounds in typical speech development. Nevertheless, it should be noted that for children with organic deficiencies (such as dysglossia) or for neurological disorders (e.g., dysarthria), they could be useful, as, in these pathologies, there are usually deficiencies in the phonoarticulatory organs, and studies in those cases pointed to an improvement with oro-motor movements [55,56].

We also attempted to clarify whether nonverbal oro-motor exercises would be more efficient for some phonemes, considering that our intervention was general and non-specific for non-produced sounds in the child’s repertoire. Despite having made an exhaustive, wide statistical analysis, analyzing phoneme by phoneme first (or by consonant groups), as well as grouping them in reference to several criteria (alveolar, fricative, all consonant groups with /l/ and with /r/, etc.) we did not find significant differences between the group that received the intervention program and the one that did not. There were differences regarding the severity of the problem: i.e., participants who had a more severe deficit (more than seven non-produced sounds) improved more than those with moderate ones (between two and six non-produced sounds), but these results were not due to intervention with the verbal oro-motor exercises as children in both groups improved in the same way.

Doubts about efficiency in this kind of intervention are based on the open debate that has lasted for over a decade between defenders of nonverbal oro-motor exercises for phonetic disorders (e.g., [12,15,36,37,54]) and authors who claim that this type of treatment is not efficient in children with typical language development (e.g., [40,41,42,43,47,57]).

In this study, as in others carried out in the English-speaking context, evidence supported the claim that nonverbal oro-motor exercises do not improve the articulation of sounds in children with typical development [13]. According to these authors, movements to produce speech are different from isolated movements of articulatory organs, and hence, there is no evidence to support the idea that these movements will improve language development. Indeed, some authors claimed that motor action neural circuits used for muscular activities or isolated movements of structures that participate in speech, such as the soft palate, tongue, or lips, are different from those that produce speech [58]. Thus, for instance, the movement of lifting the tongue inside the mouth and touching the palate is not the same movement as that which produces the phoneme /t/.

In addition, from the phonological perspective, it is assumed that phonemes are not static but vary depending on their position in the word [31]. Hence, both articulation and audition influence the child’s language development, enabling coarticulation. Thus, syllables, and not phonemes, should be considered, as well as their order in the word when analyzing phonological disorders. These are usually understood as simplification phonological processes [13]. From this perspective, auditory discrimination is considered one of the main causes of speech disorders, and it was proposed that phonemes are contrasted with other phonemes (minimum pairs) to improve phonetic–phonological difficulties [2,11,30]. It is also possible that the intervention carried out was not the most adequate for phoneme acquisition. Some authors have claimed that nonverbal oro-motor exercises should be specifically adjusted for each phoneme [54,59] and not used for a wide number. It should also be considered that a 3-month intervention might not be long enough for relevant improvements.

More empirical evidence is needed to evaluate the specific efficiency of nonverbal oro-motor exercises compared to other intervention methods such as blowing, auditory discrimination, syllable, word repetition, etc. [15]. Equally, some interventions in speech therapy are based more on tradition than on scientific foundation and should be reconsidered, as well as books that recommend the use of non-verbal oro-motor movement exercises for articulatory disorders without the necessary empirical evidence on their efficiency. Our study plays a notable preventive role, as nonverbal oro-motor exercises are widely used in preschool centers to prevent speech difficulties (see, for example, [48]), but this practice should be questioned in accordance with accumulative experimental evidence on the topic.

## Figures and Tables

**Table 1:** Distribution of participants by school and experimental and control groups.

Schools		Number	Number of Children with Difficulties in the Realization of Sounds	Moderate Difficulties	Severe Difficulties
School 1	*N*	50	24 (−1)	16 (−1)	8
Experimental G			12 (−1)	8 (−1)	4
Control G			12	8	4
Boys		28	15	10	5
Girls		22	9 (−1)	6 (−1)	3
School 2	*N*	72	31 (−2)	21	10 (−2)
Experimental G			16 (−1)	10	6 (−1)
Control G			15 (−1)	11	4 (−1)
Boys		32	17 (−1)	12	5 (−1)
Girls		40	14 (−1)	9	5 (−1)
Total		122	55 (−3)	37 (−1)	18 (−2)

Note. From the initial 55 children, 3 were eliminated, as explained in the text.

**Table 2:** Nonverbal oro-motor exercises selected for intervention.

N°	Nonverbal Oro-Motor Exercise	Articulatory Organ
1	Open mouth and closed mouth	
2	Lips making an angry position (lips to the front) and a smile position (not showing teeth)	
3	Upper lip covers lower lip and vice versa	Lips
4	Upper teeth bite lower lip and vice versa	
5	Join upper and lower teeth and show these in the mirror, i.e., opening lips wide and then hiding the teeth behind the lips	
6	Lick the upper teeth from the outer side first and from the inner side later. The same with the lower teeth	
7	Bring the tongue in and out of mouth	
8	Put the tip of the tongue at the front and back of the incisor superior teeth	
9	Put the tongue in a wide shape (between the teeth) outside the mouth and then in a narrow shape outside the mouth	
10	Stick out the tongue as far as possible, moving it up and down	
11	Move the tongue to the right and left of the corner of the mouth without it touching lips	
12	Touch the upper molar teeth with the tip of the tongue, then the lower molar teeth	
13	Move the tongue toward the right inside the mouth—as if it was a sweet—and then to the left	
14	Make a clicking sound with the tongue—like a horse trot	
15	Move the tip of the tongue along the roof of the mouth (from the hard to the soft palate) and then along the floor of the mouth (from the inferior tooth socket to the base of the mouth)	
16	Blow raspberries with lips and tongue	
17	Make the gesture of yawning and close the mouth	
18	Make the gesture of kissing noisily and smile	
19	Inflate the cheeks, release air, and then suck in the cheeks	Facial Gesture
20	Inflate the right cheek and then the left one quickly	

**Table 3:** Number of non-produced sounds for the experimental and control groups in the pretest and post-test assessment.

	Severity	Group	Mean	SD	N
Pre	2	Control	3.50	1.54	20
		Experimental	3.47	1.42	17
	3	Control	10.83	2.04	6
		Experimental	10.67	2.34	9
Post	2	Control	1.85	2.06	20
		Experimental	2.00	1.90	17
	3	Control	4.33	2.16	6
		Experimental	5.67	3.24	9

Note. Severity 2 refers to participants who had difficulties with between two and six sounds at the pretest assessment (moderate deficiency). Severity 3 refers to those participants who had articulation difficulties with more than seven sounds at the pretest (severe deficiency).

## Data Availability

The data presented in this study are available on request from the corresponding author.
